# Revisiting the Cytotoxicity of Cationic Polyelectrolytes as a Principal Component in Layer-by-Layer Assembly Fabrication

**DOI:** 10.3390/pharmaceutics13081230

**Published:** 2021-08-09

**Authors:** Ekaterina Naumenko, Farida Akhatova, Elvira Rozhina, Rawil Fakhrullin

**Affiliations:** Institute of Fundamental Medicine and Biology, Kazan Federal University, Kreml uramı, 18, 420008 Kazan, Republic of Tatarstan, Russia; akhatovaf@gmail.com (F.A.); rozhinaelvira@gmail.com (E.R.)

**Keywords:** polyelectrolytes, polycations, layer-by-layer, cytotoxicity

## Abstract

Polycations are an essential part of layer-by-layer (LbL)-assembled drug delivery systems, especially for gene delivery. In addition, they are used for other related applications, such as cell surface engineering. As a result, an assessment of the cytotoxicity of polycations and elucidation of the mechanisms of polycation toxicity is of paramount importance. In this study, we examined in detail the effects of a variety of water-soluble, positively charged synthetic polyelectrolytes on in vitro cytotoxicity, cell and nucleus morphology, and monolayer expansion changes. We have ranked the most popular cationic polyelectrolytes from the safest to the most toxic in relation to cell cultures. 3D cellular cluster formation was disturbed by addition of polyelectrolytes in most cases in a dose-dependent manner. Atomic force microscopy allowed us to visualize in detail the structures of the polyelectrolyte–DNA complexes formed due to electrostatic interactions. Our results indicate a relationship between the structure of the polyelectrolytes and their toxicity, which is necessary for optimization of drug and gene delivery systems.

## 1. Introduction

Polyelectrolytes represent a wide group of materials that contain a high level of ionizable groups along a polymeric backbone and can be anionic, cationic, or amphiphilic [1]. Water-soluble polycations are perspective materials with unique properties that are used for various technical applications, e.g., for waste water treatment, separation of oil–water emulsions, as ionic retention aid, in shampoos, and as flocculants in the paper industry [2]; they are also used in biomedical applications such as coatings, tissue engineered constructs to mimic in vivo cellular microenvironments, stem cell “niches”, and drug delivery [3]. The layer-by-layer deposition technique (LbL) introduced in the 1990s by Lvov, Decher, and Moehwald [4,5] represents a versatile approach to produce nanostructured materials with a wide range of advantages for biomedical applications due to compatibility with physiological media, capability of incorporating bioactive molecules, extra-cellular matrix components and biopolymers in the films, tunable mechanical properties, and spatio-temporal control over film organization [3,6,7,8,9,10,11,12]. Deposition of polymeric layers on the cell surface (either a membrane or cell wall) allows producing functional coatings that can protect cells from external stress impacts, such as temperature, chemical compounds, and pH changes [13,14,15]. The binding of polyelectrolytes to cells occurs due to the electrostatic interaction with a negatively charged plasma membrane [16,17]. One of the possible applications for polyelectrolyte multilayers would be to improve the bio-interface of implantable materials [18]. Thus, these polymers need to be biocompatible, nontoxic, non-immunogenic, biodegradable, have a high drug-carrying capacity, and have controlled release of the drugs at the target site. LbL offers numerous potential advantages relative to conventional methods for the incorporation and release of nucleic acids. The LbL technique allows for a precise control over the loading of DNA by controlling film thickness or the number of layers of DNA deposited during fabrication. Moreover, LbL assembly allows encapsulating of the DNA inside a biocompatible polyelectrolyte shell, where the DNA retained the natural structure of the double helix [19].

The concept of gene therapy has extensively developed for the past two decades, aimed at the treatment of various diseases by correcting the genome of the patient. Growing popularity among the global medicinal community will allow gene therapy to become an attractive market for companies and investors [20]. The target of gene therapy may be hereditary, viral, autoimmune, or oncological diseases [20,21]. The main barrier in the way of DNA entering the cell nucleus is the double nuclear envelope. Only small molecules (<40 kD, ~10 nm) can pass through the nuclear pore complex by passive diffusion [22]. Since the free plasmid DNA released after the dissociation of the complex does not have a nuclear localization signal, a very small part of the plasmid DNA will pass into the nucleus (not more than 0.1–0.001%). In addition, it has been found that about 50% of the injected DNA degrades in the cytosol as early as 1–2 h after administration.

Obviously, finding a synthetic polycationic carrier that meets all these requirements is an extremely difficult task, and therefore a search for factors that make it possible to increase the efficiency of transfection of the polymer carrier is of great interest. Here we report on a study of the in vitro cytotoxicity of a variety of water-soluble polycations considered as drug delivery systems and non-viral vectors for gene transfer in 2D and 3D cell cultures. In this study, we determined, using atomic force microscopy, the structures of the polyelectrolyte–DNA complexes that form due to electrostatic interactions.

## 2. Materials and Methods

### 2.1. Polyelectrolytes

The following cationic polyelectrolytes (Sigma-Aldrich, St. Louise, MO, USA) were used in this study: poly(acrylamide-co-diallyldimethyl-ammonium chloride) 10 wt% solution in H_2_O (P(AAm-co-DADMAC), Mw 250,000, acrylamide, ~55 wt%); poly(allylamine hydrochloride) (PAH Mw 15 kDa); PAH Mw 70 kDa; PAH Mw 900 kDa; poly(ethyleneimine) 50 wt% solution in H_2_O (PEI); poly(diallyl dimethhyl ammonium chloride) 35 wt% solution in H_2_O; (PDADMAC average Mw < 100,000 (very low molecular weight)); poly(diallyldimethhylammonium chloride) 20 wt% solution in H_2_O (PDADMAC average Mw 100,000–200,000 (low molecular weight)); poly(diallyl dimethhyl ammonium chloride) solution 20 wt% in H_2_O (PDADMAC average Mw 200,000–350,000 (medium molecular weight)); poly(diallyldimethhylammonium chloride) 20 wt% solution in H_2_O (PDADMAC average Mw 400,000–500,000 (high molecular weight)). The hydrodynamic diameters and zeta potentials of test molecules were determined by dynamic light scattering and laser Doppler velocimetry using a Zetasizer Nano ZS instrument (Malvern Panalytical Ltd., Malvern, UK). Measurements were performed under room temperature and atmospheric pressure. Milli-Q grade water was used as a solvent and concentration of all polyelectrolytes amounted to 1%.

### 2.2. Cell Culture

Human lung carcinoma cells (A549) were cultured in DMEM (Dulbecco’s Modified Eagle’s Medium) supplemented with 10% inactivated fetal bovine serum (FBS), 45 U mL^−1^ penicillin, and 45 mg mL^−1^ streptomycin in a 5% CO_2_ humidified atmosphere at 37 °C in a CO_2_ incubator.

### 2.3. Cytotoxicity Analysis

A549 cells and polyelectrolytes were introduced into the wells of 96-well plates (Nunc, Rochester, NY, USA) simultaneously. Polyelectrolytes were used as stock solutions, which were previously dissolved in cultural medium before addition of cells; the pH value of all the solutions ranged from 7.0 to 7.5. The concentrations of polyelectrolyte ranged from 5.0 to 50.0 μg per 100,000 cells. P(AAm-co-DADMAC) was added at concentrations of 5.0–1000.0 μg per 100,000 cells on the base of a previous study of cytotoxicity, which indicated the low level of toxicity of this polyelectrolyte. Further studies were performed after cultivation of cells for 24 h. The cytotoxic potential of the polyelectrolytes was estimated as changes in the activity of respiratory enzymes using an MTT test. A549 cells were plated into 96-well plates (Nunc, Roskilde, Denmark) at a density of 7000 cells per well. A Tali image-based cytometer (Invitrogen, Waltham, MA, USA) was used to determine the cell number prior to seeding. After the cultivation of cells with polyelectrolytes for 24 h, the medium was removed, 200 μL of growth medium was added to the wells, 20 μL of the stock solution of 3-(4,5-dimethylthiazol-2-yl)-2,5-diphenyltetrazolium bromide (MTT) (5 mg/mL in phosphate buffered saline) were added, and plates were incubated for 4 h at 37 °C in a 5% CO_2_ humidified atmosphere. The medium with MTT solution was removed, and the water-insoluble formazan was extracted by adding dimethylsulfoxide (200 μL per well). The optical density (OD) of formazan was measured using a microplate photometer Multiskan FC (Thermo Fisher Scientific, Waltham, MA, USA) at a wavelength of 540 nm using. The cell viability (%) related to the control (medium without polycations) was calculated by test OD/control OD × 100. The IC_50_ value was calculated as the polymer concentration that inhibits the growth of 50% of cells relative to the non-treated cells.

Lysosomal activity and intensity of active membrane transport processes were studied using the cellular absorption of Neutral Red (NR) dye. The working solution of NR (0.033%) was prepared in the appropriate medium immediately prior to the experiment. A549 cells were plated simultaneously with polyelectrolytes at various concentrations in 96-well plates at a density of 10,000 cells per well and incubated in a humidified atmosphere with 5% CO_2_ at 37 °C for 24 h. The medium was replaced with 200 μL of a working solution of NR and the plates were incubated in a CO_2_ incubator for 3 h followed by NR solution replacement with 200 μL of solvent (1% glacial acetic acid in 50% ethanol, prepared immediately before use) to extract the dye from the cells. The OD of the NR solution was measured using a microplate photometer after shaking for 15 min at room temperature at 540 (test) and 690 (reference) nm. The IC_50_ was calculated as described above for the MTT test.

To evaluate whether the cell death induced by the polyelectrolytes was due to apoptosis or necrosis, the A549 cells grown on a medium with polyelectrolytes were stained with Alexa Fluor^®^ 488 annexin V/Dead Cell Apoptosis Kit (Invitrogen, Waltham, MA, USA), according to the manufacturer recommendations. The assay was performed using a BD FACS Aria III flow cytometer (BD Bioscience, East Rutherford, NJ, USA). The apoptogenic effect of polyelectrolytes was estimated using the IC_50_ values obtained using the MTT and NR tests.

### 2.4. Cellular/Nuclear Morphology Study

The nuclear morphology of the cells was studied by staining of the A549 cells incubated for 24 h with polyelectrolytes at concentrations of 100 μg per 100,000 cells, and the IC_50_ for each polyelectrolyte with 4′,6′-diamidino-2-phenylindole (0.1 μg mL^−1^) (DAPI, Sigma-Aldrich, St. Louise, MO, USA). The specimens were stained for 5 min, followed by fixation. Optical and fluorescent images were obtained using an Axio Imager microscope (Carl Zeiss, Jena, Germany) equipped with an AxioCam HRC CCD camera. The monolayer expansion was observed during the cultivation of both polyelectrolytes treated and intact cells using an inverted Axio Observer A1 microscope (Carl Zeiss, Germany).

To assess the effect of polyelectrolytes on the cytoskeleton and cellular morphology, A549 cells grown on sterile cover slips in the cultural medium containing polyelectrolytes were stained with DAPI; cells were then washed twice with phosphate-buffered saline (PBS) and fixed with 4% paraformaldehyde for 20 min, followed by washing with PBS. Cells were permeabialized with 1% Triton X-100. To prevent any non-specific binding, a blocking solution (1% bovine serum albumin in PBS) was applied for 30 min. Specimens were stained with Alexa Fluor 488^®^ conjugated with phalloidin (Life Technologies, Carlsbad, CA, USA) according to the protocol provided by the manufacturer and mounted onto Eukitt mounting medium (Sigma-Aldrich, Saint Louis, MO, USA). The cell cultures were visualized using a confocal laser scanning microscope (LSM 780; Carl Zeiss, Germany); the images were processed using ZEN software (ZEN 3.4 (blue edition)).

### 2.5. Multicellular Spheroids Formation

Multicellular spheroids were formed from cells treated with polyelectrolytes and seeded into “handing drops”, as described previously [23]. P(AAm-co-DADMAC) was added at concentrations of 50.0, 500.0, and 5000 μg mL^−1^; and PAH Mw 70 kDa, PEI, and PDADMAC (average Mw 200,000–350,000) in concentrations of IC_50_, 1/2, and 1/5 of IC_50_.

### 2.6. Transmission Electronic Microscopy (TEM)

TEM images of the thin-sectioned cells treated with polyelectrolytes (PAH 70 kDa, PEI, PDADMAC average Mw 200,000–350,000 (IC_50_), and P(AAm-co-DADMAC) (100 µg/10^5^ cells)) were obtained using a 1200 EX microscope (JEOL, Tokyo, Japan) operating at 80 kV. The specimens were fixed with 2.5% glutaraldehyde, gradually dehydrated using a series of ethanol solutions (10–96%), embedded into Epon resin, and thin sections were cut using a ultramicrotome (LKB, Wetzlar, Germany) equipped with a diamond knife and mounted on copper grids. The thin-sectioned cells were stained using 2% aqueous uranyl acetate and lead citrate solutions.

### 2.7. Atomic Force Microscopy (AFM)

AFM images were obtained using a Dimension Icon instrument (Bruker, Billerica, MA, USA) operating in PeakForce Tapping mode [24]. To obtain the images, ScanAsyst-Air (Bruker, USA) probes (nominal length 115 μm, tip of radius 2 nm, spring stiffness 0.4 N m^−1^) was used throughout this study. The images were obtained at 512–1024 lines per scan at 0.8–0.9 Hz, allowing for the correct determination of the mechanical properties. Topography (height sensor and peak force error) and non-specific adhesion nanomechanical data were collected simultaneously. The Nanoscope Analysis software v.1.7. (Bruker, Billerica, MA, USA) was used for data processing.

### 2.8. Statistics

Each experiment was repeated at least in triplicate. All statistical analyses were performed using GraphPad Prism version 5.0 (GraphPad PRISM^®^) and Microsoft Excel 2010. Data are expressed as mean ± standard deviation. Comparisons were made using Student’s *t*-test with Welch’s correction. Statistical significance was set at 0.05.

## 3. Results

### 3.1. Determination of Hydrodynamic Size and Zeta Potential

The hydrodynamic diameters of the polyelectrolyte molecules and their zeta potentials are presented in Table 1.

As expected, all the polymers in the water are positively charged, allowing for the effective adsorption on a negatively charged cell surface (Table 1). The polymers studied here have relatively high molecular weights, therefore they form large coacervates in water, as confirmed by DLS.

### 3.2. Cytotoxic Activity of Polyelectrolytes

**Figure 1:**
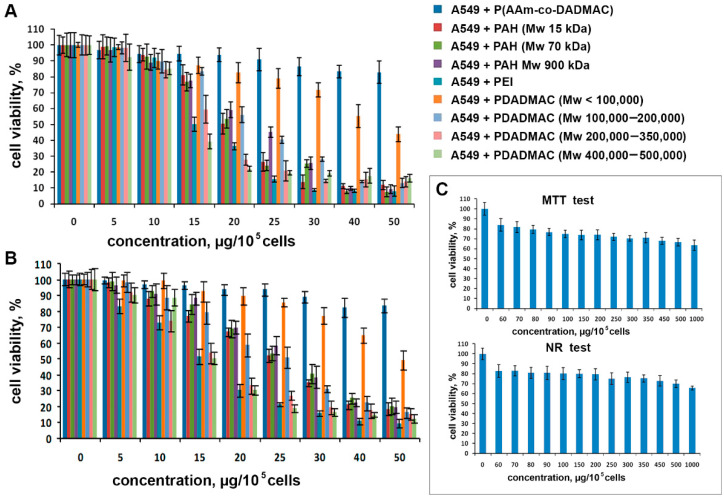
Cytotoxic effects of the cationic polyelectrolytes on A549 cells: (**A**)—MTT test; (**B**)—neutral red test. (**C**)—cytotoxic effect of P (AAm-co-DADMAC) on A549 cells.

Next, we used the MTT test to determine the effect of the polyelectrolytes on the total activity of mitochondrial dehydrogenases; the membrane transport and physiological activity of the lysosomes were tested with Neutral Red (Figure 1).

We demonstrated that all polyelectrolytes affect the metabolic activity of A549 cells in a dose-dependent manner; that is, the cytotoxicity of the cationic polyelectrolytes decreased with a decrease in concentration. The concentration of polyelectrolytes of 5 μg per 100,000 cells had no adverse effect; the survival of cells ranged from 92.4 to 99.0%. An increase in the concentration of polyelectrolytes to 15.0 μg per 100,000 cells allowed considering PEI, PDADMAC average Mw 200,000–350,000, and PDADMAC average Mw 400,000–500,000 as toxic polyelectrolytes. The lowest cytotoxic effect at a concentration of 50.0 μg was observed in the case of P(AAm-co-DADMAC), whereas PAH Mw 70 kDa demonstrated the highest toxicity level.

**Figure 2:**
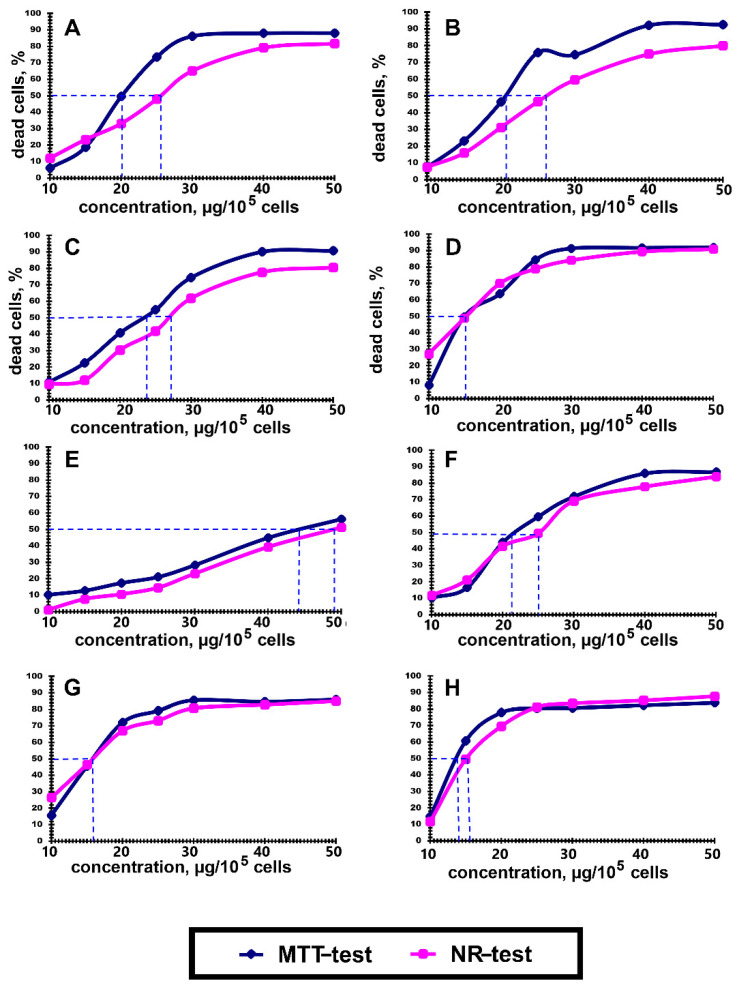
Inhibitory concentrations of polyelectrolytes, suppressing the growth of 50% of A549 cells in culture (IC_50_) under the influence of the following polyelectrolytes: (**A**)—PAH Mw 15 kDa; (**B**)—PAH Mw 70 kDa; (**C**)—PAH Mw 900 kDa; (**D**)—PEI; (**E**)—PDADMAC average Mw < 100,000 (very low molecular weight); (**F**)—PDADMAC average Mw 100,000–200,000 (low molecular weight); (**G**)—PDADMAC average Mw 200,000–350,000 (medium molecular weight); (**H**)—PDADMAC average Mw 400,000–500,000 (high molecular weight).

When studying the lysosomal activity and the intensity of the processes of active membrane transport (absorption of the neutral red dye), we observed well-matching correlations with the data obtained in the MTT test (Figure 1B). However, at the maximal concentration of almost all polyelectrolytes, the mitochondria were found to be the most sensitive structures. Due to the fact that P(AAm-co-DADMAC) has a low toxicity compared to other polyelectrolytes, a further study has been carried out, increasing its concentration to 1000 μg per 100,000 cells (Figure 1C). Thus, as the P(AAm-co-DADMAC) concentration was increased from 60.0 to 1000 μg, the cell viability in the MTT test (Figure 1C) decreased from 83.9 to 63.6%, respectively. Therefore, it was impossible to estimate an IC_50_ value for this polyelectrolyte. Figure 2 shows the IC_50_ values of all polyelectrolytes exempt for P(AAm-co-DADMAC).

Cytotoxicity assays performed in this study yielded in comparable results and allowed the following ranking of the polyelectrolytes with regard to cytotoxicity: P(AAm-co-DADMAC) < PDADMAC average Mw < 100,000 (very low molecular weight) < PAH Mw 900 kDa < PDADMAC average Mw 100,000–200,000 (low molecular weight) < PAH Mw 15 kDa < PAH Mw 70 < PDADMAC average Mw 200,000–350,000 (medium molecular weight) < PDADMAC average Mw 400,000–500,000 (high molecular weight) < PEI.

**Figure 3:**
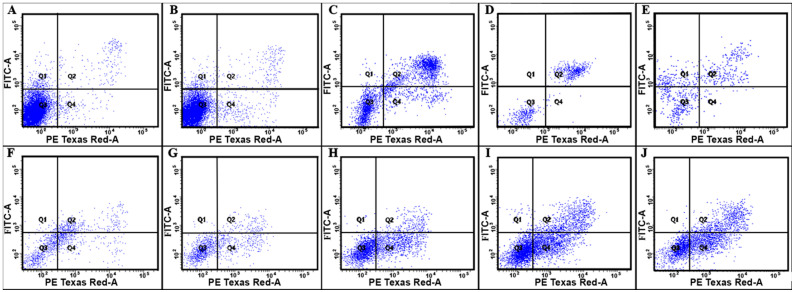
Apoptosis-inducing activity of the polyelectrolytes on A549 cells: (**A**)—Control; (**B**)—P(AAm-co-DADMAC); (**C**)—PAH Mw 15 kDa; (**D**)—PAH Mw 70 kDa; PDADMAC; (**E**)—PAH Mw 900 kDa; (**F**)—PEI; (**G**)—PDADMAC average Mw < 100,000 (very low molecular weight); (**H**)—PDADMAC average Mw 100,000–200,000 (low molecular weight); (**I**)—PDADMAC average Mw 200,000–350,000 (medium molecular weight); (**J**)—PDADMAC average Mw 400,000–500,000 (high molecular weight). Incubation time—24 h; all polyelectrolytes were introduced at IC_50_, according the results of the MTT test and the neutral red test (average value). P(AAm-co-DADMAC) was introduced at a concentration of 100 μg per 100,000 cells.

To obtain further insights into the nature of cell death and estimate the number of living, apoptotic, and necrotic (or so-called late apoptotic) cells, we used a method based on double fluorescent staining with annexin V-FITC and propidium iodide (PI) (Table 2, Figure 3).

The analysis of the apoptosis-inducing activity of the polyelectrolytes during 24 h of incubation with A549 cells showed that the proportion of cells in the apoptotic state was most pronounced in the case of PAH Mw 900 kDa (21.2%); at the same time, almost equal amount of cells were necrotic (23%). The number of living cells in the samples with P(AAm-co-DADMAC) did not have significant differences from the control, non-treated cells.

In addition, a study of the effect of polyelectrolytes on the monolayer expansion of A549 cells was carried out during 72 h of incubation by observing changes in the morphology of cells and of the cellular monolayer. All polyelectrolytes, with the exception of P (AAm-co-DADMAC), were introduced at IC_50_. P(AAm-co-DADMAC) was added at a concentration of 100 μg per 100,000 cells. The substantial changes in individual cells and monolayer morphology were detected microscopically. After 24 h of incubation, strong inhibition of cell growth was noted in all the samples except for the sample with P(AAm-co-DADMAC). In the cultures there was a large number of lysed as well as dead, detached from the bottom of the well, freely floating cells and cell debris.

**Figure 4:**
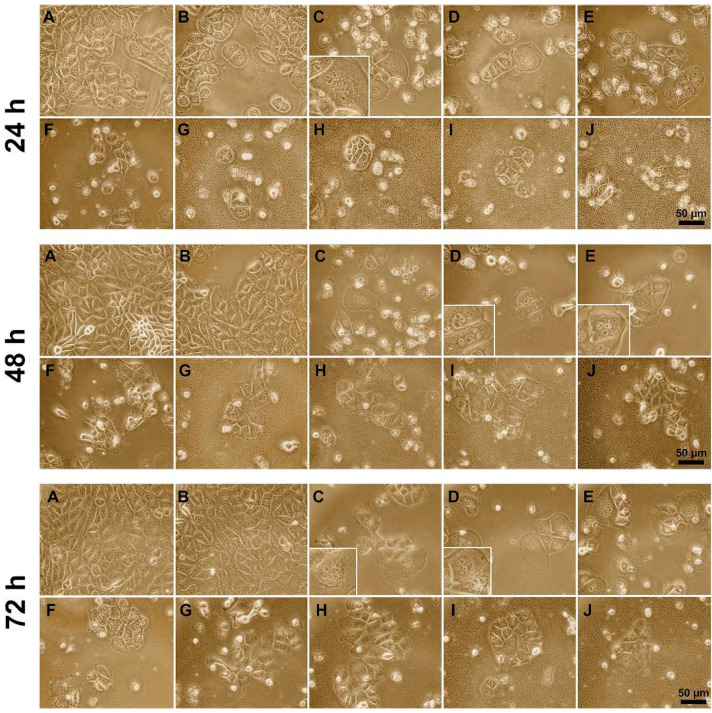
The influence of polyelectrolytes on monolayer expansion of A549 cells: (**A**)—Control; (**B**)—P(AAm-co-DADMAC); (**C**)—A549 c PAH Mw 15 kDa; (**D**)—PAH Mw 70 kDa; (**E**)—PAH Mw 900 kDa; (**F**)—PEI; (**G**)—PDADMAC average Mw < 100,000 (very low molecular weight); (**H**)—PDADMAC average Mw 100,000–200,000 (low molecular weight); (**I**)—PDADMAC average Mw 200,000–350,000 (medium molecular weight); (**J**)—PDADMAC average Mw 400,000–500,000 (high molecular weight). Inserts demonstrate multinucleated cells.

In the cells treated with PAH Mw 15 kDa and 70 kDa, a portion of the cells was strongly swelled, and some of cells contained two or more nuclei. However, after treatment with P (AAm-co-DADMAC), the morphology of the cells did not change, although there was a slight lag in growth compared to the control cells (Figure 4 24 h).

After 48 h of incubation (Figure 4) in the samples with PAH (C, D, E), it was revealed that the destructive changes are manifested as defects of cellular division and formation of syncytia (symplasts)-like cells. We also observed syncytium (symplast) with nuclei of normal size and nucleus several times smaller, indicating the transformation of nuclei towards the supercoiling of DNA (Figure 4, insets). In samples treated with other polyelectrolytes, multinucleated cells were absent; however, some cells, especially in samples treated with PEI and PDADMAC average Mw 400,000–500,000, had uneven concave contours, called cell shrinkage (decrease in cell volume).

On the third day of cultivation with polyelectrolyte P (AAm-co-DADMAC) no damage could be detected compared with the control cells (Figure 4 72 h). In other samples, the proliferative activity was weak. The morphological disorder manifested in form of changes in the shape and presence of multinucleated cells (Figure 4 72 h, inserts).

**Figure 5:**
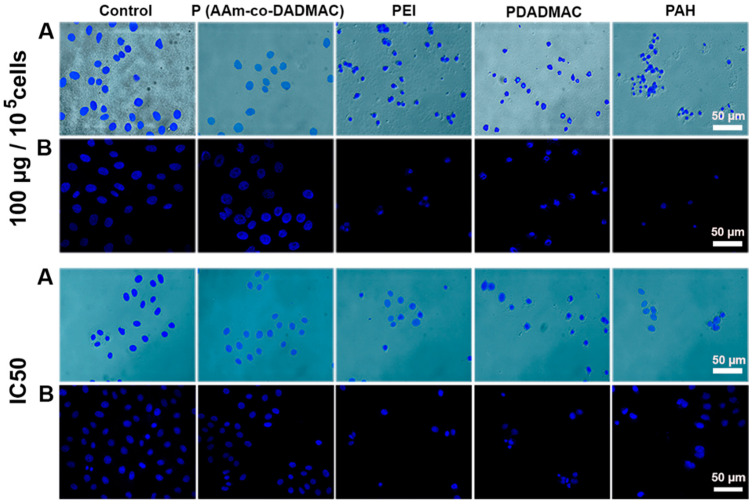
Nuclear morphology of intact and coated with various polyelectrolytes A549 cells after 24 h of cultivation. Polyelectrolytes are introduced at a concentration of 100 μg/10^5^ cells and IC_50_ (P (AAm-co-DADMAC) was introduced at a concentration of 100 μg/10^5^ cells in both cases). (**A**)—light microscopy images overlaid with fluorescence; (**B**)—confocal microscopy. Nuclei were stained with DAPI.

The nuclei of cells coated with polyelectrolyte P(AAm-co-DADMAC) have a morphology similar to that in the intact cells. In the samples treated with PEI, PDADMAC, and PAH we have observed a significant decrease in the number of nuclei with vacuolization; the chromatin was super condensed, with the shape of a half-moon (horseshoe). In the case of polyelectrolytes introduction in the IC_50_, we have observed both cells with normal nuclei and cells with fused nuclei or having a diminished nucleus size and chromatin condensation (Figure 5).

**Figure 6:**
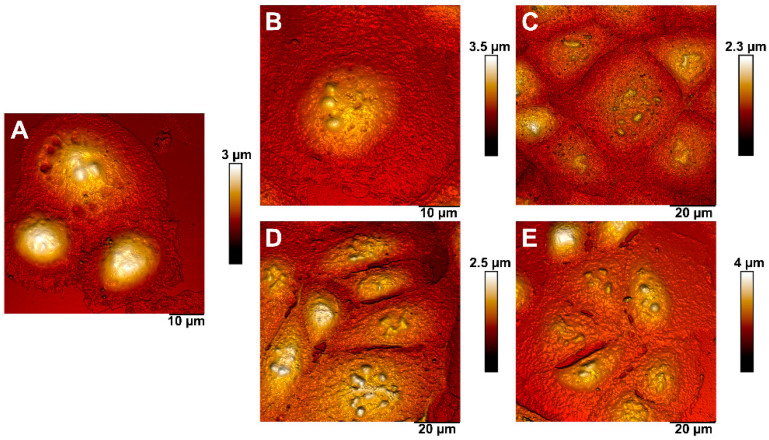
AFM images of intact and coated with polyelectrolytes A549 cells. (**A**)—control; (**B**)—P(AAm-co-DADMAC); (**C**)—PEI; (**D**)—PDADMAC average Mw 200,000–350,000 (medium molecular weight); (**E**)—PAH Mw 70 kDa.

We used AFM for the detailed visualization of cellular morphology and 3D topography (Figure 6). AFM images of the cell monolayer area allow detecting the changes in the nucleus organization in cells coated with polyelectrolytes. These changes were more pronounced in case of PEI and PAH. In the same time, the nuclear morphology of the cells coated with P(AAm-co-DADMAC was closest to the control cells. Cells coated with PEI, PAH, and PDADMAC showed changes in intercellular contacts due to the fact that they become visibly straighter and at a higher tension, which is especially pronounced in case of PEI. Gaps were observed in the cell contacts of the PAH- and PDADMAC-treated cells, which may indicate a violation of the structure of tight junction proteins.

**Figure 7:**
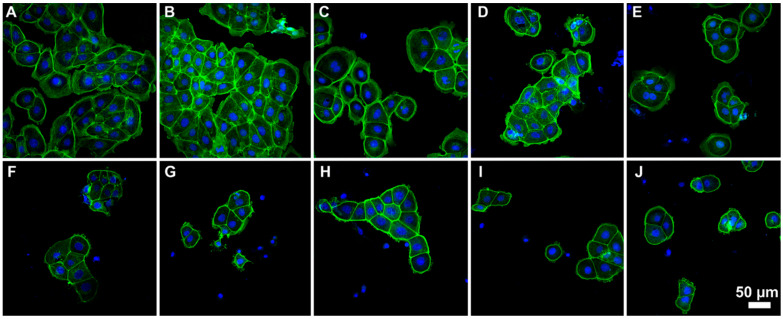
Effects of polyelectrolytes on F-actin architecture in A549 cells after 24 h of cultivation. Double staining with Alexa Fluor 488^®^ conjugate of phalloidin (green) and DAPI (blue). Polyelectrolytes were introduced at IC_50_. P (AAm-co-DADMAC) was introduced at a concentration of 100 μg/10^5^ cells. (**A**)—control; (**B**)—P (AAm-co-DADMAC); (**C**)—PAH Mw 15 kDa; (**D**)—PAH Mw 70 kDa; (**E**)—PAH Mw 900 kDa; (**F**)—PEI; (**G**)—PDADMAC average Mw < 100,000 (very low molecular weight); (**H**)—PDADMAC average Mw 100,000–200,000 (low molecular weight); (**I**)—PDADMAC average Mw 200,000–350,000 (medium molecular weight); (**J**)—PDADMAC average Mw 400,000–500,000 (high molecular weight).

Staining the cells with fluorescently-tagged phalloidin allowed us to detect the changes in the spatial organization of the actin cytoskeleton after the 24 h incubation of A549 cells with polyelectrolytes. Figure 7 shows the changes in the structure of the actin cytoskeleton of A549 cells after 24-h incubation with various polyelectrolytes.

The untreated cells, as well as the cells incubated with P(AAm-co-DADMAC) have a characteristic spatial organization of actin microfilaments beams with well-marked actin stress fibers of high tension (Figure 7). After 24 h of exposure, cells with an amorphous actin distribution in the intracellular space appeared in the experimental samples. A549 cells have lost the normal architecture of actin microfilaments after cultivation with polyelectrolytes. In polyelectrolytes samples (except for P(AAm-co-DADMAC)) there are cells with a small nucleus and a complete loss of stress fibers, as well as with a violation of the entire organization of the cytoskeleton.

**Figure 8:**
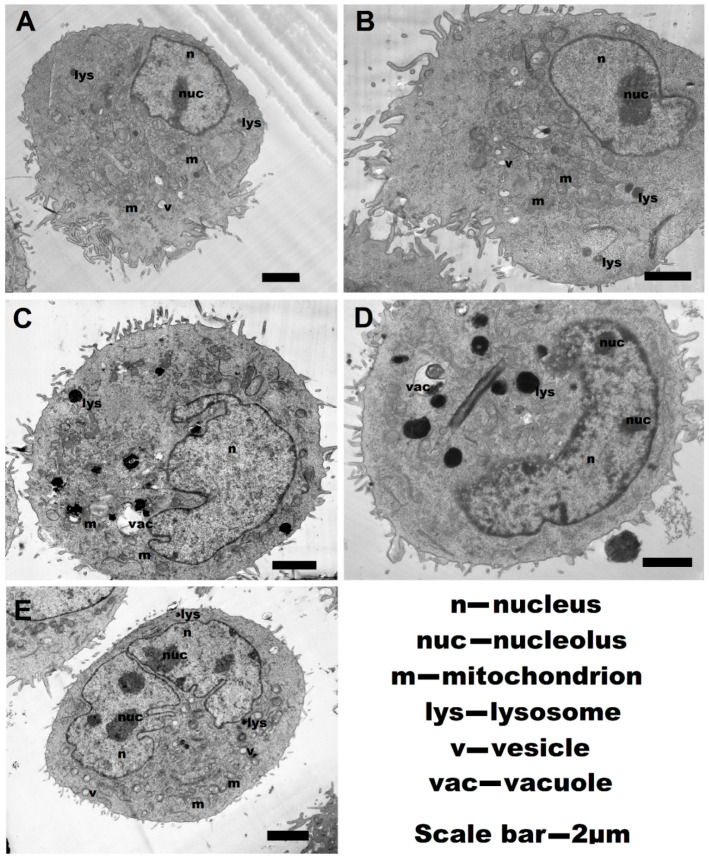
The ultrastructure of A549 cells incubated with various polyelectrolytes for 24 h: (**A**)—control; (**B**)—P(AAm-co-DADMAC); (**C**)—PDADMAC; (**D**)—PEI; (**E**)—PAH.

Data on the effect of the polyelectrolytes introduced into the IC_50_ concentration on the cell morphology were subsequently confirmed using transmission electron microscopy (Figure 8).

Dramatic changes in the ultrastructure of the cells during incubation with the polyelectrolytes were observed. Non-treated control cells were round in shape, with the apical pole containing numerous long microvilli (Figure 8A). To this pole, the organelles (mitochondria, microbodies, elements of the endoplasmic reticulum, and the Golgi complex), and the derivatives of the endomembrane system (vacuoles and vesicles), were shifted. To the opposite pole, the nucleus of the cell was displaced, containing one nucleolus in the plane of the cut. The nucleolus was visualized as a densely stained, dark structure which is associated with the nuclear membrane. The mitochondria are rounded or oval with a matrix of medium electron density and lamellar cristae, which expand the surface area of the inner mitochondrial membrane oriented across the long axis of the mitochondrion. The matrix of lysosomes is homogeneous and electron-dense. Endosomes were visualized as round-shaped, electron-transparent vesicular structures; their content is insignificant.

The general cell topography of A549 cells incubated with P(AAm-co-DADMAC) (Figure 8B) remained similar to the control; the apical and basal regions are well compromised. There were changes in the nucleus, expressed as a slight condensation of chromatin, and in the organization of the nucleoli. In the cells, the number of derivatives of the endoplasmic reticulum and the Golgi complex vesicles increased, which can be an indicator of their activation in the endomembrane system. The cavities of the endoplasmic reticulum were enlarged and often curved, which was accompanied by an increase in the number of primary endosomes in the cytosol. Electron-transparent zones were revealed in the matrix of lysosomes. Secondary endosomes contained a small amount of intracellular material, the remains of the membranes, which may indicate a moderate, rather weak stimulation of autophagy in these cells.

Morphological changes in cells treated with PDADMAC (Figure 8C) are manifested by the appearance of aggregates of the membrane-bounded derivatives of the endoplasmic reticulum in the cytosol and osmiophilic phagosomes and lysosomes. The osmiophilic substance was detected in microvilli and beyond the cell on the membrane. Many lipid drops can be visualized inside the cells.

The polarity of the cells with polyelectrolyte PEI (Figure 8D) was less pronounced than in the control samples. Significant changes in the ultrastructure of cells were observed. For example, the number of primary endosomes were enhanced, as well as the intensive proliferation of the vesicles (elements of the network and derivatives of the Golgi complex) observed. Lipid drops visualized in the cytosol can be an indicator of a change in lipid metabolism. In the cytoplasm, pleomorphic osmiophil formations containing electron-dense structures that vary in size with osmiophilic granules were detected. The latter are detected as lysosomes, endosomes, and phagosomes.

By their organization, cells treated with PAH are almost identical to cells treated with P(AAm-co-DADMAC). However, in the cytosol, there are zones containing compact accumulations of numerous membrane-bound vesicles, derivatives of the endoplasmic reticulum. Changes in the mitochondrial morphology were expressed in their swelling and matrix enlightenment.

### 3.3. Influence of Polyelectrolytes on 3D Cell Clusters Formation

The possibility to form three-dimensional cell clusters, which is a characteristic of the directed cell growth, was studied after the treatment of cells with polycations; the optical microscopy images are shown in Figure 8.

**Figure 9:**
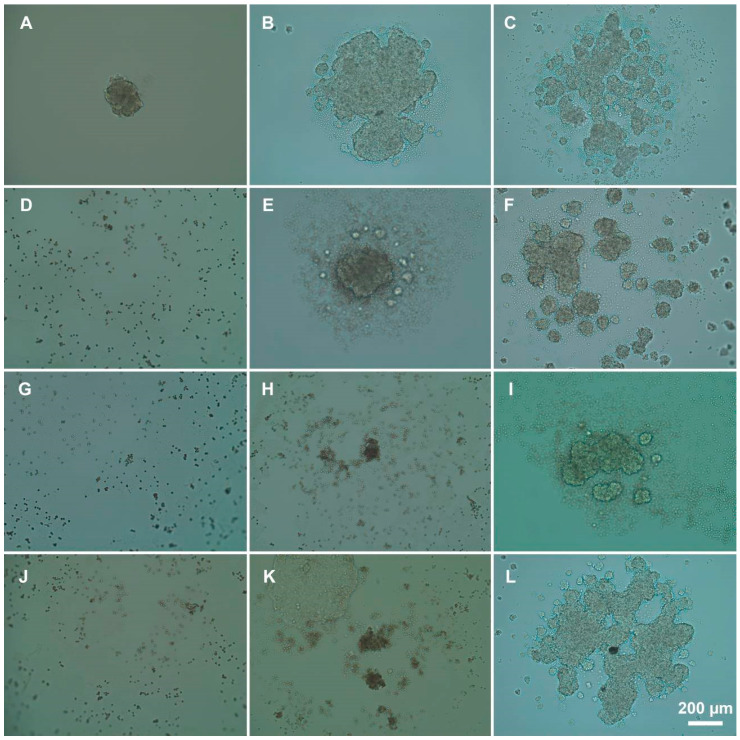
The effects of polyelectrolytes on the formation of spheroids from treated A549 cells: (**A**–**C**)—P(AAm-co-DADMAC) at 50, 500, and 5000 μg/mL, respectively; (**D**–**F**)—PAH 70 kDa, IC_50_, 1/2, and 1/5 IC_50_, respectively. (**G**–**I**)—PDADMAC average Mw 200,000–350,000 IC_50_, 1/2, and 1/5 IC_50_, respectively. (**J**–**L**)—PEI, IC_50_, 1/2, and 1/5 IC_50_, respectively.

P(AAm-co-DADMAC) does not adversely affect the formation of multicellular spheroids (Figure 9A–C). The lack of formation of multicellular structures was observed when exposed to PEI in the IC_50_ (Figure 9J). Single structures began to appear after a two-fold decrease in concentration (Figure 9K); a four-fold decrease led to the formation plenty of spheroids (Figure 9L). The addition of PAH and PDADMAC (Figure 9D–I, respectively) showed that PAH in the IC_50_ concentration interferes with the formation of the 3D clusters; as the concentration of polyelectrolyte decreases, spheroids began to form (Figure 9E,F). However, the introduction of PDADMAC, in a concentration two times less than IC_50_, has a negative effect on the formation of 3D spheroids (Figure 9H).

### 3.4. Complexation of DNA and Polyelectrolytes

**Figure 10:**
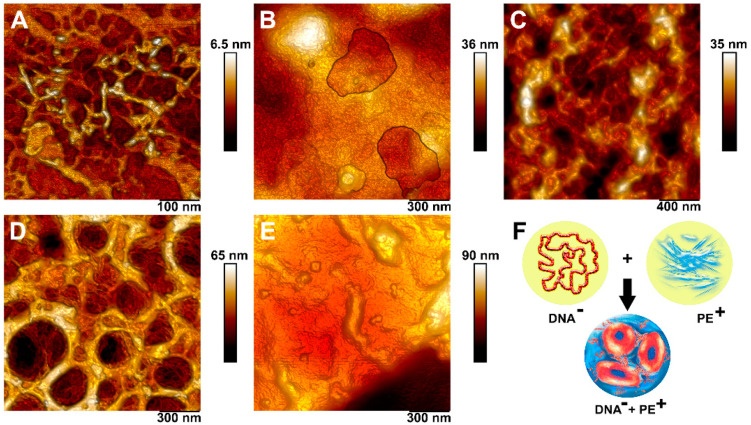
High-resolution visualization of a polycation–DNA complex: (**A**)—DNA; (**B**)—DNA+P(AAm-co-DADMAC); (**C**)—DNA+PEI; (**D**)—DNA+PDADMAC; (**E**)—DNA+PAH; (**F**)—scheme illustrating the formation of a complex of a negatively charged DNA and a positively charged polyelectrolytes. On the scheme, molecules of DNA are marked in red, and molecules of positively charged polyelectrolytes are in blue.

To visualize the complexes formed during the interaction of double-stranded DNA and polyelectrolytes, we used atomic force microscopy. The specimens were obtained by applying a dsDNA solution to the surface of a slide and then drying at room temperature to obtain a DNA sample. Similarly, samples were obtained after treating the DNA with polycations. We obtained AFM images of the DNA complexes + polycation (Figure 10). The height of the DNA molecules was about 6 nm and DNA represented a chaotic strand with a width of 2 to 4 nm. DNA + PDADMAC complexes represented the round-shaped structures, which consist of condensed DNA molecules connected to each other and molecules of the polycation, which were located between the DNA strands.

## 4. Discussion

MTT and NR tests, providing almost similar results in our study, allowed for ranking the order of toxicity of the polyelectrolytes. Generally, the MTT test is based on the ability of the succinate dehydrogenase enzyme of the mitochondrial membrane of mammalian cells to reduce the yellow salt of 3-(4,5-dimethylthiazol-2-yl)-2,5-diphenyltetrazolium bromide to violet-colored formazan crystals, accumulating as a result of this reaction in the cytoplasm of living cells [25]. The intensity of the accumulation of formazan crystals in the cytoplasm corresponds to the level of mitochondrial respiration, which is an indicator of cell viability. The amount of formazan formed inside the cells is proportional to the number of physiological active cells [26]. Neutral red is a weak cationic dye that easily penetrates cell membranes through non-diffusive, intercellular accumulation in lysosomes [27]. The change in the sensitivity of the surface of the lysosomal membrane leads to lysosomal fragility and other changes that gradually become irreversible. Such changes can be caused by exposure to various toxic substances, while reducing the absorption and binding of neutral red. Thus, it becomes possible to distinguish viable, damaged, or dead cells, which is the basis of this method.

Biocompatibility depends on the different properties of the polyelectrolytes, such as their molecular weight and charge, and on the type of cationic functionalities, structure, and sequence and conformational flexibility [28,29,30]. In our previous studies, we used polyelectrolyte-coated nanoparticles for the modification of the cell surface. The cytotoxic effects of poly(allylamine hydrochloride) were determined on human skin fibroblasts and neuronal progenitor cells, but at the same time, PAH-treated nanoparticles did not have a toxic effect on cells [31]. The biocompatibility of the polyelectrolyte-modified magnetic nanoparticles also was demonstrated in our previous studies [23,32,33]. On the other hand, we have found that polycations adsorbed onto halloysite nanotubes surfaces significantly increase the low toxicity of pristine clay nanotubes, inducing cellular and nuclear defects [34]. As for the nature of the cytotoxicity, its dependence on the molecular weight of the compound was demonstrated in this study only in the case of PDADMAC; so, with an increase in molecular weight, toxicity in relation to A549 cells increased. At the same time, PAH with a molecular weight of 900 kDa was less toxic than PAH with molecular weights of 15 and 70 kDa. An increase in cytotoxicity as a function of the molecular weight was observed for DEAE-dextran earlier [35]. The dependence of cytotoxicity on the zeta potential of polyelectrolyte molecules can also be traced in the case of PDADMAC with different molecular weights. In our study, the lowest toxicity was possessed by P(AAm-co-DADMAC), which has the lowest zeta potential and hydrodynamic diameter.

The study of the nature of cell death caused by polycations is not well covered in the available literature. Fischer et al. in their study have demonstrated that the PEI- and PEI/DNA complex-induced cell damage was not inhibited by a caspase inhibitor, indicating the necrotic type of cell death [35]. In our study, we used AnnexinV/PI double staining of cells incubated with polycations to determine the nature of cell death by fluorescence-activated cell sorting. Annexin V specifically and with high affinity binds to phosphatidylserine, which appears on the surface of apoptotic and necrotic cells; PI penetrates only cells with a compromised membrane. Apoptotic cells are stained with Annexin V alone, since they retain the integrity of the membrane in the early stages of apoptosis, while necrotic cells are stained with both reagents. Mostly in our study, cell death during cultivation with polyelectrolytes was caused by aponecrosis, a new type of cell death with incomplete execution of the apoptotic program and the following cell degeneration in necrosis [36].

Among the basic structures of the cell cytoskeleton, its actin component is most attracted by researchers, as the most labile and rapidly reacting to various effects [37]. The cytoskeleton plays key roles in the maintenance of cell architecture, adhesion, migration, differentiation, division, and organelle transport. The cellular cytoskeleton and its associated elements play a major role in the reception of various signals and their transduction into the cell [38,39]. Disruption of the cytoskeleton components and changes in spatial organization can be considered as an indicator of toxicity towards the cells, which is caused by the interaction of the charged filaments and polyelectrolytes.

Examining TEM images, we found changes in the cellular ultrastructure that may indicate a decrease in the metabolic activity of cells and moderate induction of autophagy. Thus, of all the polyelectrolytes, the effect on cell ultrastructure from the polyelectrolyte P(AAm-co-DADMAC) was the most sparing while other polyelectrolytes inflict distinct changes. The structure of the cells and their external structure in our study also changed dramatically when cells were treated with polyelectrolytes. The structure of cells and their external structure in our study also changed dramatically when cells were treated with polyelectrolytes, which was studied by atomic force microscopy. This method allowed to study in detail the change in the structure of the nuclei, which is explained by the necrotic and apoptotic pathways of cell death. In addition, changes were revealed in the structure of cellular dense junctions, which, according to data obtained in the work of Cartagena-Rivera et al., may indicate a violation of the synthesis of proteins ZO1/ZO2 [40].

In this study we used multicellular spheroids because the 3D cellular structure allows to investigate a more accurate cell response, since the conditions therein are as close as possible to those in vivo [41,42]. Such a study was carried out for the first time and requires further study of the nature of the possibility of the formation of cell clusters under the influence of polycations and their influence on the viability of cells within the cluster.

The formation of DNA–polyelectrolyte complexes is promising in the field of gene therapy. Under certain conditions in aqueous solutions, electrostatic interactions between the negatively charged phosphate groups of DNA and positively charged groups of polycations lead to the formation of compact structures. Typically, the polycation is in excess in the composition of the complexes. As a result, the complex is soluble and positively charged [43]. Furthermore, the polycation/DNA complexes were usually found to be less cytotoxic than not complexed polycations [35]. At the next stage, we visualize the formed complexes using the AFM method. Wolfert and Seymour [44] pioneered this method. In our study, we managed to obtain high-resolution photomicrographs on which the boundaries of two types of molecules at the places of their connection are clearly visible.

## 5. Conclusions

In our study, we analyzed the cytotoxicity of various polyelectrolytes and assessed the possibility of their binding to DNA. A detailed study of the ultrastructural changes in cells under the influence of polyelectrolytes has also been carried out. For the polyelectrolyte PDADMAC, a direct dependence of cytotoxicity on molecular weight and charge was confirmed. We assume that this phenomenon is determined by the level of binding to the cell membrane, depending on the charge. At the same time, no such dependence was revealed for the PAH polycation. In this study, P(AAm-co-DADMAC) demonstrated the best biocompatibility whereas PEI had the most prominent negative effect on cell viability and the formation of multicellular clusters. Based on the results obtained, it can be concluded that DNA complexes with low-molecular-weight, low-charge polycations are most preferable for use in gene therapy.

## Figures and Tables

**Table 1:** Hydrodynamic diameters and zeta potentials of the polyelectrolytes in water.

Polyelectrolyte	Size (d.nm)	Zeta Potential (mV)
Poly(acrylamide-co-diallyldimethyl-ammonium chloride) 10 wt% solution in H_2_O P(AAm-co-DADMAC)	83.9 ± 0.4	7.0 ± 0.4
Poly(allylamine hydrochloride) PAH Mw15 kDa	160.3 ± 12.2	20.1 ± 2.5
Poly(allylamine hydrochloride) PAH Mw 70 kDa	306.2 ± 32.7	38.0 ± 3.8
Poly(allylamine hydrochloride) PAH Mw 900 kDa	196.8 ± 19.4	68.9 ± 6.6
Poly(ethyleneimine) 50 wt% solution in H_2_O PEI	85.7 ± 0.7	21.1 ± 1.0
Poly(diallyldimethhylammonium chloride) solution 35 wt% in H_2_O PDADMAC average Mw < 100,000 (very low molecular weight)	120.2 ± 6.8	31.1 ± 2.3
Poly(diallyldimethhylammonium chloride) solution 20 wt% in H_2_O PDADMAC average Mw 100,000–200,000 (low molecular weight)	150.8 ± 14.8	34.0 ± 2.1
Poly(diallyldimethhylammonium chloride) solution 20 wt% in H_2_O PDADMAC average Mw 200,000–350,000 (medium molecular weight)	350.6 ± 33.6	55.3 ± 5.5
Poly(diallyldimethhylammonium chloride) solution 20 wt% in H_2_O PDADMAC average Mw 400,000–500,000 (high molecular weight)	700.8 ± 40.2	56.4 ± 4.3

**Table 2:** Apoptosis-inducing activity of the polyelectrolytes on A549 cells.

Polyelectrolyte	Parameters			
	Apoptosis, % * (Q1)	Aponecrosis, % * (Q2)	Alive Cells, % * (Q3)	Necrosis, % * (Q4)
Control	1.5	1.1	96.5	1.0
P(AAm-co-DADMAC)	1.2	1.2	96.0	1.6
PAH Mw 15 kDa	1.3	43.6	44.5	10.6
PAH Mw 70 kDa	-	56.0	42.3	1.6
PAH Mw 900 kDa	21.2	31.6	43.9	3.3
PEI	3.0	21.9	52.1	23.0
PDADMAC average Mw < 100,000	0.7	8.9	68.3	22.1
PDADMAC average Mw 100,000–200,000	0.5	7.6	59.9	32.0
PDADMAC average Mw 200,000–350,000	0.8	15.7	51.0	32.5
PDADMAC average Mw 400,000–500,000	0.6	23.9	38.5	37.0

* Representative values.
